# Study of types of some species of “*Filaria*” (Nematoda) parasites of small mammals described by von Linstow and Molin

**DOI:** 10.1051/parasite/2011182151

**Published:** 2011-05-15

**Authors:** R. Guerrero, O. Bain

**Affiliations:** 1 Laboratorio de Biologia de Vectores y Parasitos, Instituto de Zoologia y Ecologia Tropical, Universidad Central de Venezuela P.O. Box 47058 Caracas 1041A Venezuela; 2 Parasitologie comparée, UMR 7205 CNRS, Muséum National d’Histoire Naturelle 61, rue Buffon 75231 Paris Cedex 05 France

**Keywords:** *Filaria hepatica*, *Filaria circularis*, *Filaria serpicula*, *Filaria hyalina*, *Filaria vesperuginis*, *Litomosa*, *Litomosoides*, *Filaria hepatica*, *Filaria circularis*, *Filaria serpicula*, *Filaria hyalina*, *Filaria vesperuginis*, *Litomosa*, *Litomosoides*

## Abstract

Parasitic nematodes from the Berlin (ZMB) and Vienna (NMW) Museum collections referred to the genus *Filaria* Mueller, 1787 by von Linstow or Molin were studied. Three samples were in good condition and the specimens redescribed. *Litomosa hepatica* (von Linstow, 1897) n. comb., sample ZMB Vermes Entozoa 3368, from the megachiropteran *Pteropus neohibernicus*, Bismarck Archipelago, resembles *L. maki* Tibayrenc, Bain & Ramanchandran, 1979, from *Pteropus vampyrus*, in Malaysia, but the buccal capsule differs. Both species display particular morphological characters which differ from species of *Litomosa* parasitic in microchiropterans. The remaining material originates from Brazil. The spicule morphology of *Litomosoides circularis* (von Linstow, 1899) Chandler, 1931, sample ZMB Vermes Entozoa 1059 from *Hesperomys* spec. (= *Holochilus brasiliensis*), Porto Alegre, confirms that it belongs to the *sigmodontis* group; the microfilaria presents characters of the genus *Litomosoides*, *e.g.* body attenuated at both extremities and salient cephalic hook. Taxonomic discussions by others confirm that species of *Litomosoides* belonging to the *sigmodontis* group and described subsequently are distinct from *L. circularis*. *Litomosoides serpicula* (Molin, 1858) Guerrero, Martin, Gardner & Bain, 2002, is redescribed, sample NMW 6323 from the bat *Phyllostoma spiculatum* (= *Sturnira lilium*), Ypanema. It is very close to *L. brasiliensis* Almeida, 1936, type host *Moytis* sp., but distinguished by a single ring in the buccal capsule, rather than two, supporting previous conclusions that the taxon *L. brasiliensis*, as generally regarded, may represent a complex of species. Samples NMW 6322 and NMW 6324, from other bats and also identified by Molin (1858) as *Filaria serpicula*, contain unidentifiable fragments of *Litomosoides incertae sedis*. *Filaria hyalina* von Linstow, 1890, sample ZMB Vermes Entozoa Q 3905 from *Sorex vulgaris* (= *Sorex araneus*), is *incertae sedis* because it contains two unidentifiable posterior parts of male, which might be an acuarid, *Stammerinema* sp. *Filaria vesperuginis* von Linstow, 1885, sample ZMB Vermes Entozoa Q 3929, from the bat *Vesperugo serotinus* (= *Eptesicus serotinus*), contains encysted nematode larvae and is a *nomen dubium*.

## Abstract (fr)

Étude de nématodes des collections des Muséums de Berlin (ZMB) et de Vienne (NMW) attribués au genre *Filaria* Mueller, 1787 par von Linstow ou Molin. Trois lots sont en bon état et les spécimens redécrits. *Litomosa hepatica* (von Linstow, 1897) n. comb., lot ZMB Vermes Entozoa 3368, parasite du mégachiroptère *Pteropus neohibernicus*, Archipel Bismarck, ressemble à *L. maki* Tibayrenc, Bain & Ramanchandran, 1979, de *Pteropus vampyrus*, en Malaisie, mais la capsule buccale est différente. Les deux espèces ont des caractères morphologiques particuliers qui les distinguent des *Litomosa* parasites de microchiroptères. Les autres matériels sont originaires du Brésil. La morphologie des spicules de *Litomosoides circularis* (von Linstow, 1899) Chandler, 1931, lot ZMB Vermes Entozoa 1059 d’*Hesperomys* spec. (= *Holochilus brasiliensis*), Porto Alegre, confirme que cette espèce appartient au groupe *sigmodontis ;* la microfilaire présente les caractères du genre *Litomosoides*, corps effilé aux deux extrémités et crochet céphalique saillant. Une discussion taxonomique confirme que les espèces de *Litomosoides* du groupe *sigmodontis* décrites ultérieurement sont distinctes de *L. circularis*. *Litomosoides serpicula* (Molin, 1858) Guerrero, Martin, Gardner & Bain, 2002, est redécrit, lot NMW 6323 chez le microchiroptère *Phyllostoma spiculatum* (= *Sturnira lilium*), Ypanema. Elle est très proche de *L. brasiliensis* Almeida, 1936, hôte type *Moytis* sp., mais distincte par la capsule buccale avec un anneau cuticulaire, au lieu de deux, ce qui renforce les conclusions précédentes indiquant que le taxon *L. brasiliensis*, tel qu’il est généralement utilisé, représente vraisemblablement un complexe d’espèces. Les lots NMW 6322 et NMW 6324, d’autres microchiroptères et aussi identifiés par Molin (1858) à *Filaria serpicula*, contiennent des fragments non identifiables de *Litomosoides incertae sedis*. *Filaria hyalina* von Linstow, 1890, lot ZMB Vermes Entozoa Q 3905 de *Sorex vulgaris* (= *Sorex araneus*), est *incertae sedis* parce qu’il contient deux régions postérieures de mâle qui pourraient être un acuaride, *Stammerinema* sp. *Filaria vesperuginis* von Linstow, 1885, lot ZMB Vermes Entozoa Q 3929, du microchiroptère *Vesperugo serotinus* (= *Eptesicus serotinus*), contient des larves de nématodes enkystées et est un *nomen dubium*.

## Introduction

*Filaria* Mueller, 1787 was used generally for thread-like nematode parasites found in body cavities of hosts (Stiles & Hassall, 1922). The genus was later split into different genera distinguished by characters which often had not been considered in the original descriptions (Anderson & Bain, 2009). Analyzing ancient type material is useful to clarify their taxonomic positions. We studied here types of five species of “*Filaria*” (seven samples) parasitic in small mammals and described by von Linstow (1885, 1890, 1897, 1899) and Molin (1858). None have been re-observed since, although two have previously been allocated to a more recent filarioid genus, *Litomosoides* Chandler, 1931 (Chandler, 1931; Guerrero *et al.*, 2002).

## Material and Methods

Von Linstow’s types are from the Collection Vermes of the Museum für Naturkunde der Humboldt-Universität in Berlin (ZMB) and those of Molin are preserved in the Collection of Evertebrata Varia at the Naturhistorisches Museum Wien (NMW).

Morphology and terminology of filarioids follow Bain (1966) and Guerrero *et al.* (2002). Specimens were cleared in lactophenol and drawn with a microscope equipped with a camera lucida. Particular attention was given to head papillae (the inner circle corresponds to the external labial papillae, and the external circle to the cephalic papillae), buccal capsule and *area rugosa* of the posterior region of the male. When possible, the male tail was placed in ventral view to assess the number and arrangement of caudal papillae: for a given pair, papillae are placed on a transverse line or they are more or less aligned on the mid-ventral line. If sufficient females were available, microfilariae were extracted from their uteri. ND indicates that the particular measurement was not determined for that specimen.

Measurements were taken from drawings and are in micrometres, unless otherwise stated. When hololectotype and allolectotype were designated their measurements appear first, followed by the measurements of paralectotypes in parentheses.

Regarding the hosts, the original name is given first followed by present name which is consistent with Wilson & Reeder (2005). The identification of the typehost of *Filaria circularis* von Linstow, 1899 presented a particular problem which was solved with the collaboration of the curator of the mammalian collection in Berlin. The type-host specimen, nro 1050, from Porto Alegre, had been identified by Hensel (1872) as *Hesperomys*? sp. Species of *Hesperomys* are at present contained in several genera (Musser *et al.*, 1998) and, ealier, Hershkovitz (1955) had transferred specimens of *Hesperomys* from Porto Alegre to *Holochilus*. The type-host specimen itself is not in the Berlin collection but the eight “*Hesperomys*” specimens collected by Hensel in Porto Alegre and stored in the Berlin collection were identified to *Holochilus brasiliensis* (Desmarets, 1819) by Alfred L. Gardner when he revised the collection during his visit in Berlin (1978) and this identification is validated by the current curator Dr Frieder Mayer. Thus the type host of *F. circularis* was very likely *H. brasiliensis*.

## Morphological Analysis of the Type Materials and Taxonomic Consequences

Three samples contain whole specimens well preserved and were redescribed. The other four samples contain fragment of specimens not identifiable or specimens that do not fit with the original description.

### 1. *Litomosa hepatica* (von Linstow, 1897) n. comb.

Sample: ZMB Vermes Entozoa 3368.

Host: *Pteropus neohibernicus* (Megachiroptera: Pteropidae).

Location: bile ducts.

Locality: Bismarck Archipelago, West Pacific.

Type specimens: hololectotype (a female; ZMB Vermes Entozoa 3368) and two female paralectotypes, one complete, one without caudal region (ZMB Vermes Entozoa 7428).

**Fig. 1.**
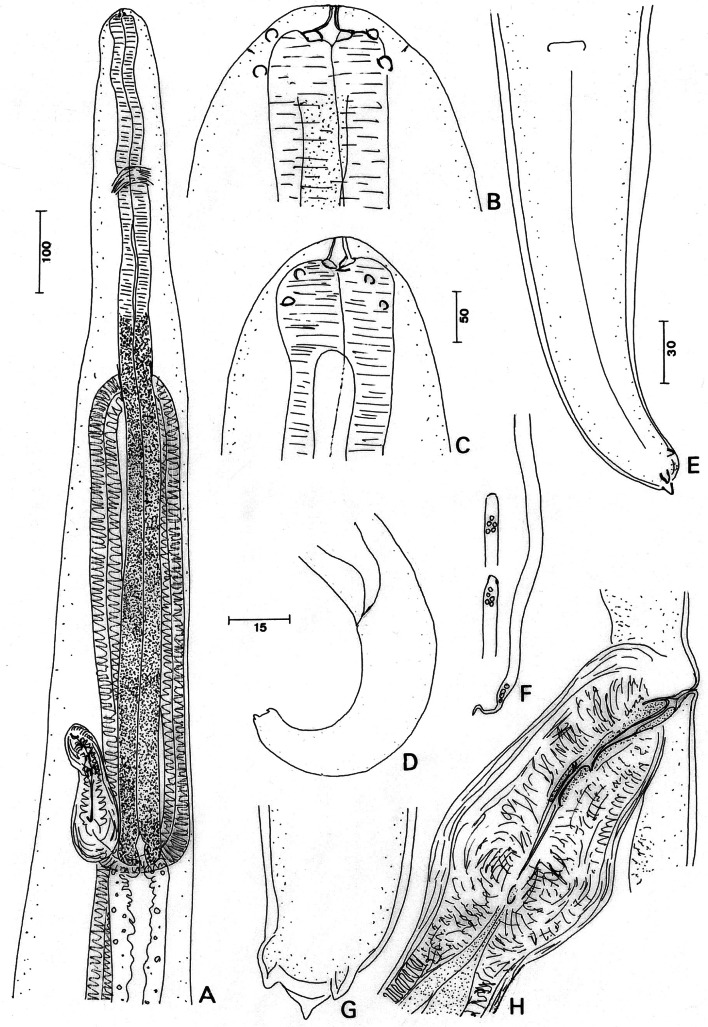
*Litomosa hepatica* (von Linstow, 1897) n. comb. (= *Filaria hepatica*). Female: A. Anterior part, subventral view; B. Head, dorsoventral view (ventral chord figured and dotted); C. Head, lateral view (internal cuticular lateral crest figured); D. Tail, lateral view; E. Tail, ventral view; F. Uterine microfilaria, anterior part (lateral view above, ventral view below) and posterior part on right; G. Another female, caudal extremity, ventral view; H. Vagina, lateral view. Scales in μm: A, 100; B, C, F, G, 15; D, 50; E, H 30.

General (Fig. 1): female body medium-sized, tapered at both ends. Head attenuated, rounded. Four externolabial papillae and four cephalic papillae in two rectangular cephalic shields expanded laterally, amphids tiny (Fig. 1B, C). Buccal capsule slightly shorter than broad; anterior part with thin wall and posterior thicker ring embedded in oesophagus. Oesophagus with anterior muscular and posterior glandular parts of similar diameter. Vagina elongated, straight; ovejector directed anteriorly in its first half (Fig. 1A, H). Tail robust (Fig. 1D, E), extremity more or less truncated with three or four terminal lappets, one dorso-apical, one ventral and two lateral (Fig. 1E, G).

Measurements (female hololectotype and paralectotypes): length 43.8 (39.3) mm, maximum width 367 (323 and 333), at the nerve ring 81 (78 and 108), at oesophageal-intestinal junction 224 (185 and 212), at vulva 196 (169 and 184). Buccal capsule 10 (8 and 8) long and 12 wide; buccal capsule height/width ratio 0.83 (0.67 and 0.67). Nerve ring to anterior end 213 (196 and 203). Oesophagus 1,136 (955 and 1,062) long, 55 (51 and 52) wide. Vulva 894 (932 and 991) to apex. Vagina 152 (164 and 173) long, 57 (63 and 81) wide; ovejector 1,450 long, 43 wide. Tail 261 (280) long; width at anus 77 (82).

Microfilaria (from ovejector, two entire specimens and several in pieces): body straight 128 and 135 long, maximum width 3.5-4.5, sheath not identified; anterior end slightly attenuated in dorso-ventral view, but not in lateral view; tiny cephalic hook; cephalic space longer than wide; last caudal nucleus far from tail tip, distal part of tail thin, undulated (Fig. 1F).

#### • Taxonomic discussion

These female specimens resemble those of *Litomosa maki* Tibayrenc, Bain & Ramachandran, 1979 described from *Pteropus vampyrus* (Linnaeus, 1758) in Malaysia (Ramachandran *et al.*, 1966; Tibayrenc *et al.*, 1979). Both have a complete set of head papillae (four externo-labial and four cephalic papillae), situated rather far from apex, a long oesophagus (≥ 1 mm) but a relatively small buccal capsule, and a truncated tail extremity with conspicuous conical lappets. No male was in the present sample but von Linstow (1897) gave the description translated here: “The 33.4 mm long and 0.31 mm wide male is distinctly narrowed posteriorly and the tail end is coiled in two close turns; the oesophagus is 1/22.8 (1,465 mm), the tail is 1/169 (0.198 mm) of the whole length; the spicules are very unequal, measuring 0.18 and 0.078 mm; at the tail end there are three teeth, one dorsal and two latero-ventral; papillae are not apparent”. The characters of the male confirm the resemblance with *L. maki*. However, von Linstow’s material differs in that the buccal capsule is composed of two segments (three in *L. maki*), the oesophagus is divided, the bodies of both sexes are longer (39.3-43.8 mm and 33.4 mm, in female and male, respectively, compared to 35 mm and 25 mm, in *L. maki*, as corrected by Tibayrenc *et al.*, 1979). We thus propose the new combination *Litomosa hepatica* (von Linstow, 1897) n. comb.

The microfilaria of *L. maki* has not been described. In *L. hepatica* the body of the microfilaria is not folded as is usual in species of *Litomosa* (Petit, 1980; Guerrero *et al.*, 2002; Martin *et al.*, 2006; Junker *et al.*, 2009). In fact, the two species from megachiropterans display several morphological particularities not found in species of *Litomosa* parasitic in microchiropterans. Based on the long oesophagus, the arrangement of caudal papillae and the simple shape of the right spicule, Martin *et al.* (2006) suggested that *L. maki* from megachiropterans represented a primitive line in *Litomosa*. This is further supported by the number (8) and arrangement (far from mouth) of head papillae, in both species.

### 2. *Litomosoides circularis* (von Linstow, 1899) Chandler, 1931

Sample: ZMB Vermes Entozoa 1059.

Host: *Hesperomys*? spec. Hensel (= *Holochilus brasiliensis* Desmarest, 1819) (Rodentia: Cricetidae: Sigmodontinae).

Location: not given.

Locality: Porto Alegre, Brazil.

Type specimens: hololectotype (male; ZMB Vermes Entozoa 1059), allolectotype (female), five male and two female paralectotypes (ZMB Vermes Entozoa 7429). Other paralectotypes (one male, a male posterior part, one female, and a female anterior part with microfilariae) in MNHN Paris collection, accession number 2 JW.

**Fig. 2.**
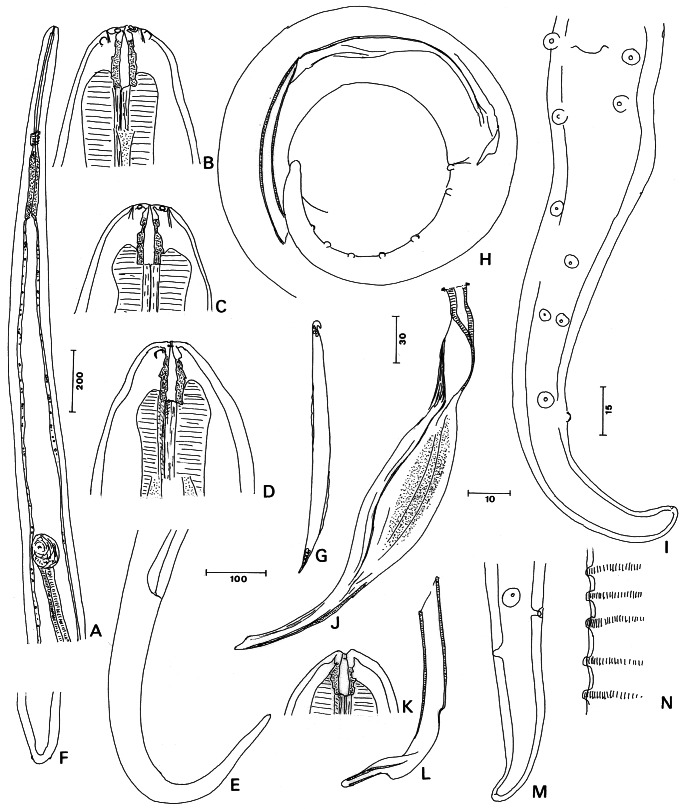
*Litomosoides circularis* (von Linstow, 1899). A-G, female: A. Anterior part; B. Head, ventral view (ventral chord figured); C. Head, dorsal view; D. Head, left lateral view (lateral chord and crest figured); E. Tail, lateral view; F. Caudal extremity, ventral view; G. Sheathed uterine microfilaria. H-N, male: H. Caudal part, lateral view; I. Tail, ventral view, except distal extremity in lateral view; J. Lamina of left spicule, sublateral view; K. Buccal capsule; L. Right spicule, lateral view; M. Caudal extremity, ventral view; N. *Area rugosa*, at mid-length, lateral view. Scales in μm: A, 200; B, C, D, I, J, K, L, M, 15; E, 100; F, G, N, 10; H, 30.

General (Fig. 2): body very attenuated at both ends in both sexes. Males 1⁄3 length of females. Head rounded, head papillae grouped near apex, asymmetrically arranged, four small externo-labial papillae, two larger ventral cephalic papillae posterior to amphids. Oral opening tiny. Buccal capsule markedly longer than broad, posterior part embedded in oesophagus; irregular external aspect, faint asymmetrical or symmetrical ring in some specimens (Fig. 2D, K). Oesophagus divided into muscular and glandular parts, of equal lengths. Nerve ring located at mid-length of oesophagus.

Male: four or five pairs of cloacal papillae, most anterior pair in adcloacal position; papillae of third and fifth pairs not symmetrically arranged (one papilla more posterior, placed on the median ventral line), papillae of fourth pair in transverse line but close to median ventral line; phasmids far from posterior end (Fig. 2I, M). Extremity of tail rounded. Lamina of left spicule with enlarged membranous part, terminal third rod-like with beveled extremity (Fig. 2H, J). Right spicule slightly sclerotized, with distal part elongated, supported by two fine cuticular rods and membranous extremity (Fig. 2L). *Area rugosa* composed of transverse bands of longitudinal crests.

Measurements (hololectotype and extremes of six paralectotypes): total length 24.1 (21.5-24.6) mm, maximum width 149 (115-149), at nerve ring 58 (47-58), at oesophageal- intestinal junction 61 (54-61). Buccal capsule 16 (14-16) long, 8 wide; buccal capsule ratio 2.0 (1.8-2.0). Nerve ring to anterior end 214 (136-230). Oesophagus 488 (454-556) long, 27 (24-33) wide. Tail 177 (119-198) long, width at cloaca 48 (45-51). *Area rugosa* from 452 (393-487) to 1,777 (1,507-1,915) from cloacal aperture, transverse ridges ND (1.0-1.4) high, distance between bands ND (6.3-12.3). Left spicule 294 (250-311) long, handle 139 (127-154) long, lamina 155 (123-180) long. Right spicule 102 (98-121) long. Spicular ratio 2.82 (2.44-2.86).

Female: post-oesophageal vulva, subspherical vagina; tail very long, straight or bent ventrally, attenuated at end.

Measurements (allolectotype and extremes of four paralectotypes): length 75.8 (59.9-80.8) mm, maximum width 254 (193-278), at the nerve ring 87 (75-88), at oesophageal-intestinal junction 88 (81-115), at vulva 136 (119-163). Buccal capsule 19 (15-20) long and 8 (8-9) wide; buccal capsule ratio 2.1 (1.7-2.5). Nerve ring to anterior end 278 (378-441). Oesophagus 617 (546-662) long, 30 (23-34) wide. Vulva 1,383 (1,098-1,806) to apex. Vagina 106 (95-106) long, 88 (81-95) wide. Tail 607 (544-633) long; width at anus 64 (59-81).

Microfilaria (from ovejector; n = 2): body fusiform 62 and 63 long, 4 wide; sheath present, as long as the microfilaria; head with protruding cephalic hook.

#### • Taxonomic discussion

The species described by von Linstow (1899) was assigned to the genus *Litomosoides* by Chandler (1931). Later Bain *et al.* (1989) distinguished two morphological groups in the genus, a *sigmodontis* and a *carinii* group. They placed von Linstow’s species in the *sigmodontis* group, despite the original description of the spicules being unclear (see von Linstow’s measurements and figure 73). The characters of the left and right spicules of von Linstow’s type material as described above confirms that *L. circularis* belongs to the *sigmodontis* group.

The morphological features that distinguish *L. circularis* from the 17 other species of the *sigmodontis* group subsequently described are listed in the following discussions (Mazza, 1928; Caballero, 1939 & 1947; Esslinger, 1973; Muller, 1980; Bain *et al.*, 1980 & 1989; Brant & Gardner, 1997; Notarnicola *et al.*, 2000 & 2002; Guerrero *et al.*, 2002; Bain *et al.*, 2003; Notarnicola, 2005; Notarnicola & Navone, 2009; Notarnicola *et al.*, 2010).

The single species of the sigmodontis group from marsupials, *L. barretti*Muller, 1980 is distinguished by a shorter buccal capsule (12 μm, measured in figure 1 of Muller), more cylindrical female tail, symmetrical arrangement of caudal papillae and higher spicular ratio, 3.0:1 (2.5:1 in *L. circularis*).

Three species are parasites of bats. *L. leonilavazquezae*Caballero, 1939 is shorter than *L. circularis* in all measurements, except the buccal capsule; *L. fosteri*Caballero, 1947 has a buccal capsule with two thickened rings, a shorter right spicule and higher spicular ratio (4.8 compared to 2.44-2.86 in *L. circularis*); *L. teshi*Esslinger, 1973 has an asymmetrical buccal capsule and longer microfilariae 75-109 (compared to 62-63 in *L. circularis*).

Thirteen species are parasites of rodents. *L. hoplomyis*Esslinger, 1973 in Eumysopinae (Echimyidae) is a very small species, 10-13 mm and 18-30 mm long respectively in males and females (21-25 mm and 60-80 mm long in *L. circularis*, respectively) and the female tail has a conical terminal part. In *L. ctenomyos*Brant & Gardner, 1997 from Ctenomyidae, the oesophagus is longer and undivided in the female and the caudal papillae are symmetrically arranged along the male tail to the tip.

The other species are parasites of Sigmodontinae (Muridae). *L. patersoni* (Mazza, 1928) redescribed by Notarnicola *et al.* (2010), has shorter, stout microfilariae (34-44), a pair of precloacal papillae, complete set of head papillae, and straight female tail with pointed extremity. Five species have prominent amphids; three of these have microfilariae longer than 75 (62-63 long in *L. circularis*): in *L. anguyai*Notarnicola, Bain & Navone, 2002 the male has a pair of precloacal papillae; *L. legerae*Bain, Petit & Berteaux, 1980 has a complete set of head papillae, a buccal capsule with thick irregular walls and microfilaria with a caudal filament; in *L. oxymycteri*Notarnicola, Bain & Navone, 2000 the fourth pair of caudal papillae is joined on the median ventral line; *L. nasuti*Notarnicola & Navone, 2009 has no apparent cephalic papillae, a buccal capsule with conspicuous ring at mid-length and phasmidial knobs; *L. navonae*Notarnicola, 2005 has six or seven pairs of caudal papillae. Three species have a buccal capsule with a symmetrical annular thickening with its posterior rim oriented backwards and some other distinctive characters: in *L. galizai*Bain, Petit & Diagne, 1989 the buccal capsule is thinner in both sexes and longer in the male, 25-30 (14-16 in *L. circularis*); in *L. khonae*Bain, Petit & Diagne, 1989 the female tail is curved dorsally and in both sexes the tail tip is acute; in *L. chagasfilhoi*Moraes Neto, Lanfredi & de Souza, 1997 the oesophagus is undivided and the left spicule has a long membranous terminal sheath. *L. sigmodontis*Chandler, 1931 has smaller and regularly arranged caudal papillae, and longer microfilariae 84.5 ± 2.9 (Bain *et al.*, 1989). *L. esslingeri*Bain, Petit & Diagne, 1989 has a straight female tail with conical end and microfilariae in a large sheath.

*Litomosoides circularis* is also distinct from the three species which are known only from females and thus cannot be assigned to the *sigmodontis* or *carinii* group. *L. solarii*Guerrero *et al.*, 2002 has a microfilaria with a sharp caudal point, and some distinctive adult characters (long undivided oesophagus, vulva in oesophageal region, thick tail). *L. chitwoodi*Bain, Guerrero & Rodriguez, 2003 has a shorter buccal capsule, 12 long. *L. artibei*Esslinger, 1973 described from a female anterior part and briefly redescribed with both sexes by Cuartas-Calles & Munoz-Arango (1999)has a longer buccal capsule, 33-34.

Two species are known only from microfilariae (Marinkelle & Garcia-Castañeda, 1999): that of *L. colombiensis*Esslinger, 1973 is twice as long and tapers posteriorly to form a narrow tail tip while that of *L. caliensis*Esslinger, 1973 has a thicker tail with rounded extremity.

In conclusion, no species of *Litomosoides* falls into synonymy with *L. circularis*.

### 3. *Litomosoides serpicula* (Molin, 1858) Guerrero, Martin, Gardner & Bain, 2002

Molin (1858) described the species from three different hosts and two localities. However during examination it appeared that only one sample, NMW 6323, was adequate for study, whereas the other two samples were small fragments of females.

Sample: NMW 6323.

Host: *Phyllostoma spiculatum* (= *Sturnira lilium*) (Chiroptera: Phyllostomidae).

Location: abdominal cavity.

Locality: Ypanema, Brazil.

Type specimens: hololectotype male, allolectotype female, a male and a female paralectotypes, three anterior and one posterior female paralectotypes.

**Fig. 3.**
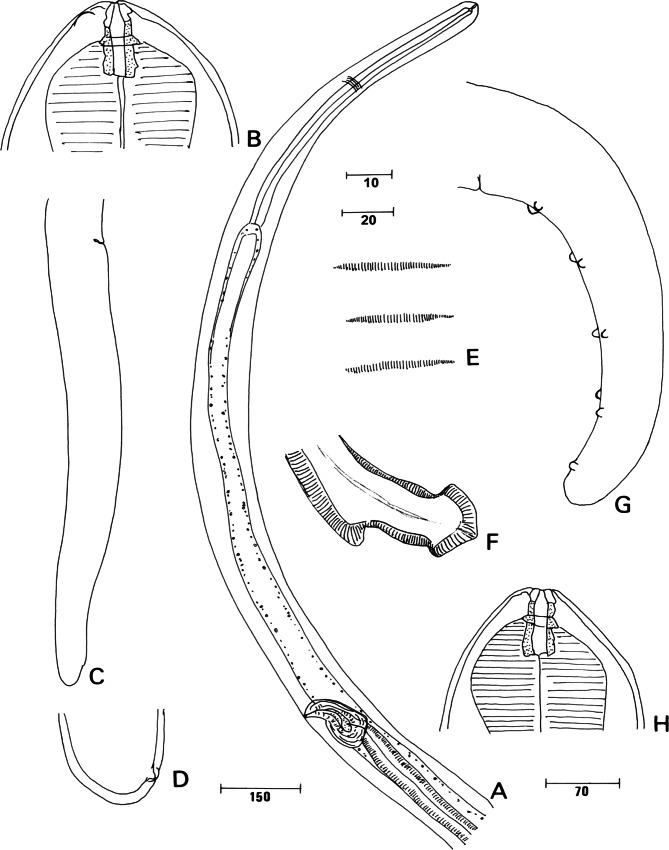
*Litomosoides serpicula* (Molin, 1858), sample NMW 6323. A-D, female: A. Anterior part, left lateral view; B. Head and buccal capsule (note a cephalic papilla); C. Tail, right lateral view; D. Other female, caudal extremity, right lateral view. E-H, male: E. *Area rugosa*, ventral view; F. Distal extremity of right spicule, lateral view; G. Tail left lateral view; H. Head and buccal capsule. Scales in μm: A, 150; C, 70; B, D, E, F, H, 10; G, 20.

General (Fig. 3A-H): large filariae, females longer than males. At present, worms brownish, and head papillae not all observed (*e.g.* external-labial papillae) but, in lateral view, two large latero-ventral cephalic papillae identified. Buccal capsule markedly longer than broad, posterior half embedded in oesophagus; thickened ring at mid-length, at level of apex of oesophagus. Division of oesophagus into muscular and glandular parts distinct or not. Nerve ring located at mid-length of oesophagus. Tail hardly attenuated.

Male: caudal papillae identified in lateral view: papillae of three anterior pairs on transverse lines, posteriorly three papillae longitudinally aligned. Right spicule with distal portion well sclerotized to its extremity, conspicuous dorsal keel posteriorly directed, subterminal rim and terminal cap (Fig. 3F).

Measurements (hololectotype and a paralectotype): Total length 39.9 and 40.3 mm, maximal width 114 and 100 at mid-body, at oesophageal-intestinal junction 67. Buccal capsule 16 long, 9 wide; buccal ratio 1.88 and 1.78. Nerve ring to anterior end 196. Oesophagus 569 and 566 long, muscular anterior part 182 long. Left spicule partly visible, length of blade 240. Tail slightly bent ventrally, 207 long, 52 wide at cloaca. *Area rugosa* from 377 to 2,150 anterior to cloacal aperture, each band of ridges 24-26 long, distance between bands 11.1-12.6, ridges 3-4 high.

Female: vulva well posterior to oesophago-intestinal junction. Vagina elongated, with distinct bulbous muscular part. Caudal end rounded, with a pair of phasmids.

Measurements (allolectotype and extremes of a complete paralectotype, and three anterior and one posterior fragments of paralectotypes): total length 61.6 (78.1) mm, maximal width at mid-body 178 (169-185), at base of oesophagus 70, at vulva 105. Buccal capsule 18 (16-21) long, 9 (9-10) wide; ratio 1.95 (1.77-2.07). Nerve ring 358 to anterior end. Length of oesophagus 699 (639-812), width 26-29. Vulva 1,772 (1,362-2,478) to apex. Vagina 134 (108-157) long, 60 wide; ovejector longer than 1,250, 41 wide. Tail 315 (431) long, 52 (57) wide at anus.

#### • Taxonomic discussion

*Filaria serpicula*Molin, 1858 was not mentioned until Guerrero *et al.* (2002) briefly observed the original specimens and found that these had the characteristic well sclerotized and segmented buccal capsule of *Litomosoides*, and resembled *L. brasiliensis*Almeida, 1936 with the pronounced distal cap and dorsal keel of the right spicule (Almeida, 1936). The taxon *L. brasiliensis*, originally described from *Myotis* sp. in Brasil, was subsequently assigned to specimens from diverse microchiropterans from South American geographic areas. However the redescriptions (Rego, 1961; Diaz-Ungria, 1963; Esslinger, 1973; Guerrero *et al.*, 2002) were not all similar, particularly in the morphology of the buccal capsule and, when studied in ventral view, the arrangement of caudal papillae (Diaz-Ungria, 1963; Guerrero *et al.*, 2002). Consequently, Guerrero *et al.* (2002) suggested that the taxon *L. brasiliensis* was very likely a complex of species and they prefered not to place *L. brasiliensis* in synonymy with the oldest taxon, *L. serpicula*.

The present study of Molin’s specimens revealed a distinct buccal capsule: a single ring (Fig. 3B, H), instead of one small anterior and one larger posterior, as described for *L. brasiliensis* by Almeida (1936), Diaz-Ungria (1963), Esslinger (1973), Guerrero *et al.* (2002) for specimens from *Carollia perspicillata*, and Notarnicola *et al.* (2010). This difference supports our previous conclusions that *Litomosoides serpicula* and *L. brasiliensis*, both described from Brasil, are two distinct species, with *Sturnira lilium* and *Myotis* sp. as respective type hosts. Guerrero *et al.* (2002) studying specimens from Peruvian *S. lilium* had noted and illustrated a single ring in the buccal capsule, or a second very small and distant anterior one. However, to decipher the question of species diversity and host specificity, morphology of microfilariae will be useful as well as molecular analysis as has been done recently for some species of *Litomosoides* (Ferri *et al.*, 2009). In addition we note here that the taxon *Filaria spiculatum* in the checklist of Ubelaker *et al.* (1977) is a *lapsus calami* for *Filaria serpicula*.

The sample NMW 6322, from the abdominal cavity of the phyllostomid bat *Phyllostoma brevicaudum* (= *Carollia brevicauda*), from Ypanema, Brazil, is composed of one anterior extremity and two fragments of female, in very poor condition. The sample NMW 6324, from the abdominal cavity of *Phyllostoma* sp. *Incerta* (sic) Rio Muria, Brazil is composed of pieces of females (one anterior extremity, another one without buccal capsule, two posterior extremities, and three fragments). Both specimens belong to *Litomosoides* but are species *incertae sedis*.

### 4. *Filaria hyalina* von Linstow, 1890 *incertae sedis*

**Fig. 4.**
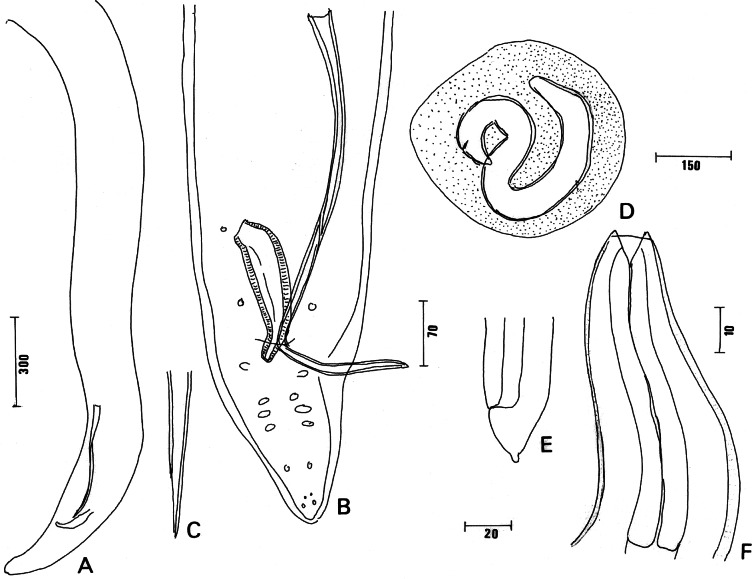
Two von Linstow samples. A-C, *Filaria hyalina* von Linstow, 1890: A. Male posterior region, left lateral view; B. Ventral view of caudal region; C. Left spicule, distal extremity, lateral view. D-F, unidentified nematode larva (= *Filaria vesperuginis*): D. Encysted larva; E. Posterior end, lateral right view; F. Anterior part. Scales in μm: A, 300; B, E, 70; C, 20; F, 10; D, 150.

The sample ZMB Vermes Entozoa Q 3905, from the intestine of *Sorex vulgaris* (= *Sorex araneus*) (Insectivora: Soricidae), probably in Europe, is composed of two males without anterior part. The ventral view of male (Fig. 4) is as figured by von Linstow. Length of the longer male piece 5.97 mm, maximum width 241 and 245; at oesophago-intestinal junction 248. Glandular oesophagus 1,950 long, 146 wide. Tail 274 and 203 long, width at cloaca 138 and 132. Left spicule 518 and 534 long, 15 and 16 wide, pointed at posterior extremity. Right spicule robust 156 and 162 long, 45 and 47 wide (Fig. 4A-C). Caudal papillae: two or three precloacal pairs, six postcloacal pairs arranged as figured (Fig. 4B); phasmids anterior to last pair of papillae. The material may be a species of *Stammerinema* Osche, 1955, a common acuarid in Soricidae, but no sound identification can be made in the absence of the characteristic dilated anterior part (Tiner, 1951; Soltys, 1951; Osche, 1955; Quentin, & Wertheim, 1986). *Filaria hyalina* is *incertae sedis*.

### 5. *Filaria vesperuginis* von Linstow, 1885 *nomen dubium*

The sample ZMB Vermes Entozoa Q 3929, from intestine of the vespertilionid bat *Vesperugo serotinus* (= *Eptesicus serotinus*), probably from Europe, is composed of five encysted larvae which are not in good condition (Fig. 4D-F). They are 874 (813-1005) long, 66 (54-81) wide; head with two projections; buccal cavity funnel-shaped 34 long; undivided oesophagus 125 long; tail 50 (34-62) long, 39 (27-59) wide at cloaca, with a terminal knob (Fig. 4D-F). We did not observe the caudal terminal small spines described and drawn by von Linstow (1885). With the undivided oesophagus, the larvae are not those commonly found in bats, such as *Spirocerca lupi* (Rudolphi, 1809) and *Physocephalus sexalatus* (Molin, 1860) (Barus and Tenora, 1970). *Filaria vesperuginis* von Linstow, 1885 is a *nomen dubium*.
